# *APOL1* variant alleles associate with reduced risk for opportunistic infections in HIV infection

**DOI:** 10.1038/s42003-021-01812-z

**Published:** 2021-03-05

**Authors:** Ping An, Efe Sezgin, Gregory D. Kirk, Priya Duggal, Elizabeth Binns-Roemer, George Nelson, Sophie Limou, Mark L. Van Natta, Douglas A. Jabs, Michelle Estrella, Jeffrey B. Kopp, Cheryl A. Winkler

**Affiliations:** 1 Basic Research Laboratory, Molecular Genetic Epidemiology Section, Basic Science Program, Frederick National Laboratory for Cancer Research, Frederick, MD USA; 2 Department of Epidemiology, the Johns Hopkins Bloomberg School of Public Health, Baltimore, MD USA; 3 Laboratory of Nutrigenomics and Epidemiology, Izmir Institute of Technology, Izmir, Turkey; 4 Department of Medicine, the Johns Hopkins University School of Medicine, Baltimore, MD USA; 5 Center for Cancer Research Informatics Core, Leidos Biomedical Research, Inc., Frederick National Laboratory for Cancer Research, Frederick, MD USA; 6 CRTI UMR1064, Inserm, Université de Nantes & ITUN, CHU Nantes, Nantes, France; 7 Ecole Centrale de Nantes, Nantes, France; 8 Department of Ophthalmology, the Wilmer Eye Institute, the Johns Hopkins University School of Medicine, Baltimore, MD USA; 9 Kidney Health Research Collaborative, Department of Medicine, University of California San Francisco, San Francisco, CA USA; 10 San Francisco VA Health Care System, San Francisco, CA USA; 11 Kidney Disease Section, National Institute of Diabetes and Digestive and Kidney Diseases, NIH, Bethesda, MD USA

**Keywords:** Genetic association study, Molecular medicine

## Abstract

Apolipoprotein L1 (APOL1), an innate immune factor against African *trypanosoma brucei*, inhibits HIV-1 in vitro. The impact of *APOL1* G1-G2 variants on HIV-1-associated opportunistic infections (OIs) is unknown. Here, we report findings from a metaanalysis of four HIV/AIDS prospective cohorts (ALIVE, LSOCA, MACS, and WIHS) including 2066 African American participants. Using a global test combining all four cohorts, carriage of two *APOL1* variant alleles is associated with a 50% reduction in odds of OI (combined OR 0.50, 95% CI 0.33-0.76). Subgroup analysis of OI etiological categories (viral, parasitic, fungal and Mycobacterial) suggests the possibility of specific protection from fungal infections (OR 0.54. 95% CI 0.32-0.93; *P*_Bonferroni corrected_ = 0.08). We observe an association of *APOL1* variant alleles with host protection against OI in HIV-positive individuals. The study suggests a broader role of *APOL1* variant alleles in innate immunity in vivo.

## Abstract (web summary)

An et al. determine the presence of variants of the innate immune factor APOL1 in four cohorts of HIV-positive patients. The study suggests that APOL1 might confer carriers of two variant alleles protection from HIV related opportunistic infections, especially fungal infections.

## Introduction

Apolipoprotein L1 (APOL1) is a human innate immune factor that is active against African trypanosomes responsible for African trypanosomiasis (sleeping sickness)^1^. Two common *APOL1* coding alleles, termed G1 and G2, are strongly associated with chronic kidney disease in African-ancestry populations. African Americans carrying two copies of *APOL1* G1 or G2 kidney risk alleles (referred to herein as *APOL1* variant alleles) have a sevenfold increased risk for nondiabetic end-stage kidney disease and a 17-fold increased risk for focal segmental glomerulosclerosis, respectively^2–4^. The strongest associations have been reported for HIV-associated nephropathy, with odds ratio (OR) 29 in African Americans and OR 89 in South Africans^3,5^, suggesting a strong interaction between APOL1 and HIV.

APOL1 is a trypanolytic protein that confers innate resistance to African *Trypanosoma brucei*^1^. Individuals with the G2 allele are resistant to acute *T.b. rhodesiense* Human African trypanosomiasis (HAT) but experience faster progression of chronic *T.b. Gambiense* HAT^6^. G1 is associated with asymptomatic carriage and undetectable parasitemia in individuals with chronic *T.b. gambiense* infection^6^. Both forms of HAT may have led to the selection of the *APOL1* renal risk alleles in Africa^2^. APOL1 also provides resistance against *Leishmania*^7^. It has recently been reported that APOL1 restricts HIV infection in macrophages and differentiated monocytes in vitro^8^. The proposed mechanisms include degradation of HIV Gag protein and depletion of HIV Vif, but the in vivo consequences are largely unknown^8^.

In addition to its role in innate immunity against trypanosomes, overexpression of the APOL1 variant in monocytes induces differentiation into macrophages, an important component of innate immunity^9^. We thus hypothesized that APOL1 could potentially confer resistance to a broad spectrum of pathogens. *APOL1* risk variants are found only on African chromosomes and are not present in European or Asian populations, except by African admixture (e.g., African Americans and Afro-Caribbeans). G1- and G2-combined allele frequencies are ~34% in African Americans and 10–50% in Sub-Saharan African populations^4^, where HIV infection is highly prevalent. Opportunistic infection (OI), caused by a variety of pathogens (bacteria, viruses, fungi, or protozoa), frequently occurs in patients with HIV infection due to a weakened immune system.

In this study, we explored a possible influence of *APOL1* variants on opportunistic infections in African Americans from four HIV/AIDS cohorts. We observed that carriage of two *APOL1* variant alleles was associated with a reduced risk of OI occurrence. Subgroup analysis of OI etiological categories (viral, parasitic, fungal, and mycobacterial) revealed a tendency of specific protection against fungal infection. Our results suggest that *APOL1* variant alleles may confer host protection against OI in HIV-positive individuals.

## Results

### Baseline characteristics in the MACS, WIHS, and LSOCA cohorts

The genotype distribution of *APOL1* genotypes was in concordance with Hardy–Weinberg equilibrium expectations in each of four cohorts (*P* > 0.05). The HIV-related characteristics of HIV-seroprevalent African Americans at study entry in a part of the ALIVE cohort, the LSOCA, MACS, and WIHS cohorts, stratified by *APOL1* genotype status, are presented in Table 1. *APOL1* variant alleles were present in 8.4, 8.3, 14.4, and 10.5% of HIV-positive participants in these cohorts, respectively.

**Table 1:** Baseline characteristics of seroprevalent participants with HIV infection at study entry by *APOL1* variant allele counts in the ALIVE, WIHS, MACS, and LSOCA cohorts.

Cohort	*APOL1* variant allele	Sample size	Male sex, %	Age (SD)	ART use, %	CD4 T-cell counts (SD)	HIV load, log_10_ (SD)
ALIVE^a^	1 or 0	196 (91.6%)	74.5	41.1 (6.1)	25.7	558.5 (278.9)	4.0 (0.92)
	2	18 (8.4%)	83.3	43.4 (3.1)	23.1	669.5 (296.0)	3.66 (0.97)
	*P* value		0.41	0.12	0.73	0.15	0.18
LSOCA	1 or 0	719 (91.7%)	67	42.6 (9.0)	82	227 (212)	3.3 (1.4)
	2	65 (8.3%)	71	43.9 (7.4)	94	227 (215)	3.2 (1.4)
	*P* value		0.52	0.5	0.02	0.98	0.49
MACS	1 or 0	557 (85.6%)	100	35.5 (8.4)	72.4	573.7 (359.0)	2.99 (1.29)
	2	94 (14.4%)	100	34.3 (8.3)	72.3	485.2 (316.2)	2.98 (1.31)
	*P* value		1.0	0.22	1.0	0.68	0.97
WIHS	1 or 0	913 (89.5%)	0	36.3 (8.0)	16.5	448.9 (297.3)	3.78 (1.14)
	2	107 (10.5%)	0	35.2 (7.4)	15	485.2 (316.2)	3.80 (1.05)
	*P* value		1.0	0.16	0.67	0.24	0.82

*SD* standard deviation, *ART* antiretroviral therapy.

Shown are the *APOL1* variant allele (1 or 0 variant allele; 2 variant alleles) distributions, sex distribution, age, rates of antiretroviral drug use (ART), CD4 T-cell counts (cells/ µL), and plasma HIV load.

*P* values were from a Chi-squared test for categorical comparisons and a *t* test for continuous variable comparisons.

^a^For seroprevalent subjects only.

Baseline HIV viral loads were not statistically different between the *APOL1* high-risk and low-risk groups in the ALIVE, LSOCA, MACS, and WIHS cohorts (*P* = 0.18, 0.49, 0.97, and 0.82, respectively, Table 1). Baseline CD4+ T-cell counts were also not statistically different between the *APOL1* high-risk and low-risk groups in the seroprevalent subgroup of the ALIVE cohort, and the seroprevalent LSOCA, MACS, and WIHS cohorts (*P* = 0.15, 0.98, 0.68, and 0.24, respectively, Table 1).

### Impact of *APOL1* variant alleles on opportunistic infections (OIs)

We evaluated whether *APOL1* variant alleles might be protective against opportunistic infections. In ALIVE (*n* = 440), carriage of two *APOL1* variant alleles (3.9% in OI+ vs. 12.1% in OI−) was recessively associated with a decreased risk of OIs (OR = 0.29, *P* = 0.040; OR_adj_ = 0.32, *P*_adj_ = 0.044, Table 2).

**Table 2:** Association of *APOL1* G1 and G2 variant alleles with opportunistic infections in HIV-positive individuals in the ALIVE cohort.

*APOL1* variant allele	OI−, *n* = 363 (%)	OI+, *n* = 77 (%)	OR (95% CI)	*P*
0	142 (39.1)	35 (45.5)	Ref.	1
1	177 (48.8)	39 (50.7)	0.89 (0.54–1.48)	0.67
2	44 (12.1)	3 (3.9)	0.28 (0.08–0.94)	0.034^a^
Recessive			0.29 (0.08–0.97)	0.040^a^
(2 vs. 1 or 0)			0.29 (0.09–0.98)	0.044^b^
Additive 2 vs. 1 vs. 0)			0.69 (0.47–1.03)	0.07^b^

Shown are the rates of opportunistic infection (OI) among subjects with carriage of 2 and 1 or 0 *APOL1* variant alleles. HIV-positive individuals included both seroconverters (*n* = 226)^11^ and seroprevalent (*n* = 214, see Table 1). The additive model approach statistical significance and the recessive model reached statistical significance.

^a^Fisher’s exact test;

^b^Adjusting for proportions of African ancestry using the first five principal components (PC), HIV-1 viral load, and age in logistic regression.

**Fig. 1:**
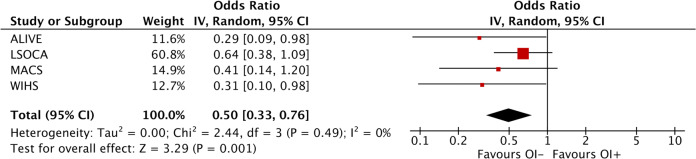
Forest plot showing the odds ratios (OR) of opportunistic infection (OI) for the carriage of 2 versus 1 or 0 *APOL1* variant alleles in the meta-analysis of four HIV/AIDS cohorts. Data were pooled from the four studies using inverse variance (IV) (Table 3). The red block indicates the point estimate of OR, while the horizontal line depicts 95% CI of OR in each study. The area of the block indicates the weight assigned to that study in the meta-analysis.

We next validated this finding in three other independent HIV-seroprevalent cohorts (LSOCA, MACS, and WIHS) by conducting a meta-analysis of the *APOL1* variant allele’s effect on opportunistic infections. In the validation cohorts, carriage of two *APOL1* variant alleles (8.7% in OI+ vs. 14.2% in OI−) was recessively associated with 36% lower odds of OI (combined OR 0.64, 95% CI 0.45–0.91, *P* = 0.006, Table 3). We also performed a meta-analysis of *APOL1* variant alleles’ effect on OI combining all four HIV cohorts (Table 3). The global test combining the four independent cohorts revealed a significant association of carriage of two *APOL1* variant alleles with protection against OI in both unadjusted analysis (OR 0.56, 95% CI 0.39–0.81, *P* = 0.002) and in an adjusted meta-analysis using covariates (including age, sex, ART usage, and HIV viral load at baseline) (OR 0.50, 95% CI 0.33–0.76, *P* = 0.001, Table 3, Fig. 1). The heterogeneity test revealed no apparent heterogeneity among cohorts (*P*_q-statistic_ ≥ 0.49). The leave-one-out meta-analysis removing any one cohort out did not abolish significance (Table 3 and Supplementary Table 1).

**Table 3:** Association of carriage of 2 versus 1 or 0 *APOL1* variant alleles with opportunistic infection in four HIV cohorts, recessive model.

Cohort	*APOL1* 2 variant alleles carriers/O+, *N* (%)	*APOL1* 2 variant alleles carriers/OI−, *N* (%)	*APOL1* 2 variant alleles carriers OR_raw_ (95% CI)	*P*_raw_	*APOL1* 2 variant alleles carriers OR_adjusted_ (95% CI)	*P*_adjusted_	Other factors among tested predicting of OI OR (95% CI), *P* OR (95% CI), *P*	
							ART	HIV viral load
ALIVE	3/77 (3.9)	44/363 (12.1)	0.29 (0.08–0.97)	0.040^a^	0.29 (0.087–0.980)^a^	0.046^a^	2.03 (1.10–3.72), 0.023	1.61 (1.12–2.32), 0.011
LSOCA	34/485 (7.0)	31/299 (10.4)	0.65 (0.39-1.09)	0.09^b^	0.65 (0.38–1.09)^b^	0.10^b^	1.27 (0.83–1.94), 0.26	1.14 (1.02–1.28), 0.019
WIHS	19/160 (11.9)	7/32 (21.9)	0.48 (0.18–1.26)	0.0.13^c^	0.31 (0.096–0.977)^c^	0.046^c^	0.04 (0.005–0.29), 0.0017	1.76 (1.04–2.97), 0.036
MACS	15/135 (11.1)	79/516 (15.3)	0.58 (0.29–1.17)	0.13^d^	0.41 (0.14–1.20)^d^	0.10^d^	0.22 (0.12–0.39), 0.0001	1.68 (1.22–2.31), 0.0014
Meta-analysis^e^	71/857 (8.3)	161/1190 (13.5)	0.56 (0.39–0.81)	0.002	0.50 (0.33–0.76)	0.001	0.50 (0.14–1.72), 0.27	1.45 (1.12–1.89), 0.006
Meta-analysis w/o ALIVE	68/780 (8.7)	117/827 (14.2)	0.60 (0.41–0.88)	0.008	0.64 (0.45–0.91)	0.006	0.28 (0.05–1.44), 0.13	1.42 (1.03–1.96), 0.03

Note: Covariates available from each cohort were used for adjustment in the logistic regression model:

^a^Adjusted for age, sex, ART, PCA 1–5, and HIV viral load.

^b^Adjusted for ART, age, sex, and HIV transmission routes; PCA not available.

^c^Adjusted for age, ART, OI, PCA 1–5 from GWAS data, HIV viral load.

^d^Adjusted for age, ART, OI prevention, HIV viral load; PCA not available.

^e^Meta-analysis using RevMan 5 with a random-effects model; heterogeneity test: *P*_q-statistic_ ≥0.49, *I*^2^ = 0.0%, for raw or adjusted analysis. For other leave-one-out metaanalyses, see Supplementary Table 1.

Among covariates tested (age, sex, ART treatment history, *Pneumocystis* pneumonia prophylaxis usage, HIV viral load, and African ancestry), HIV viral load was a consistent significant risk predictor of OI in all cohorts (OR 1.45, 95% CI 1.12–1.89), while ART usage was a protective predictor of OI in two cohorts though short of significance in the meta-analysis of four cohorts (OR 0.50, 95% CI 0.14–1.72, Table 3). These results are largely consistent with the expectation, attesting to the validity of our model testing.

### Impact of *APOL1* variant alleles on fungal infections

To determine if the APOL1 association was due to a specific OI etiology, we used the LSOCA cohort, which enrolled only participants with an AIDS diagnosis. Dichotomizing specific OI etiologies from the LSOCA cohort revealed that *APOL1* variant alleles were associated with a lower risk of fungal OI (OR = 0.54, *P* = 0.02), and not with viral, parasitic, or bacterial OIs (Table 4). With a Bonferroni correction of four categories of OIs tested, a trend of fungal protection remained (*P*_Bonferroni corrected_ = 0.08) for this exploratory analysis.

**Table 4:** Association of *APOL1* variant alleles with baseline AIDS-defining opportunistic infections among LSOCA participants under additive and recessive genetic models.

	Total OI	Additive model		Recessive model (HR)	
Outcome	*N*	OR^f^_adj_	*P*^f^_adj_	OR^f^_adj_	*P*^f^_adj_
Any viral OI^a^	161	0.91 (0.68–1.23)	0.54	0.98 (0.46–1.93)	0.95
Any parasitic OI^b^	27	0.86 (0.68–1.08)	0.20	0.77 (0.44–1.34)	0.36
Any fungal OI^c^	412	0.78 (0.63–0.98)	0.03	0.54 (0.32–0.93)	0.02
Any mycobacterial OI^d^	152	1.17 (0.87–1.57)	0.31	1.06 (0.54–2.10)	0.87
Any OI^e^	546	0.87 (0.69–1.09)	0.22	0.66 (0.40–1.11)	0.11

Shown are the associations of particular opportunistic infections (OI) with carriage of *APOL1* variant alleles for the additive (2 vs. 1 vs. 0) and recessive (2 vs. 1 or 0) genetic models.

^a^Viral opportunistic infections include any CMV, Kaposi sarcoma-related herpes virus, and herpes simplex virus.

^b^Parasitic opportunistic infections include cerebral toxoplasmosis infections, cryptosporidiosis, isosporiasis, and extrapulmonary pneumocystosis.

^c^Fungal opportunistic infections include *Pneumocystis carinii* pneumonia (PCP), candidiasis, cryptococcosis, histoplasmosis, and coccidioidomycal infections.

^d^Bacterial opportunistic infections include mycobacterium tuberculosis, *Mycobacterium avium* complex (MAC), and other mycobacterial infections.

^e^Subjects with at least one opportunistic infection of any class.

^f^Adjusted for ART, age, sex, and HIV transmission routes.

Next, to know which individual pathogens are influenced by the *APOL1* variants, we performed an explanatory analysis of the top seven most common OIs (*N* ≥ 40) in the LSOCA cohort. We saw consistent *APOL1* variant protective effect trends on fungal OIs, no effect on viral OIs, and possibly increased risk for bacterial pneumonia (OR = 2.54, *P* = 0.03) (Supplementary Table 2). There is some evidence that variant *APOL1* confers partial protection against multiple fungal pathogens, with nonsignificant protective trends for *Pneumocystis carinii* pneumonia and esophageal candidiasis and a significant protective association with Cryptococcal meningitis (*P* = 0.03). None of the 47 patients with two *APOL1* variant alleles had Cryptococcal meningitis infections. However, we note that these associations did not reach the Bonferroni-corrected significance threshold (0.05/7 = 0.007).

Last, we assessed the association of *APOL1* on multiple OI infections (or co-infections). Patients had an average of 2.16 OI diagnoses, most frequently among *Pneumocystis carinii* pneumonia, esophageal candidiasis, herpes simplex, mycobacterial, and cryptococcal infections (Supplementary Table 3). Using a multivariate regression model, we found that carriage of two copies of *APOL1* G1–G2 alleles significantly reduced the number of multiple infections (1.59 vs. 2.17 multiple infections, beta = −0.58, *P* = 0.03, adjusted for HAART, age, sex, HIV transmission route, and HIV load, Supplementary Table 3).

In the MACS cohort, there was a nonsignificant trend of protection from fungal OI (OR 0.37, 95% CI 0.12–1.14, *P* = 0.084) and PCP (OR 0.28, 95% CI 0.06–1.28, *P* = 0.10). The other cohorts, which were smaller, did not have enough subgroup outcomes for meaningful analyses.

## Discussion

*APOL1* variants have a profound impact on African-ancestry populations in predisposing to a spectrum of progressive kidney diseases, most markedly in those with untreated or undertreated HIV infection^3,10^. In this study, we assessed the influence of *APOL1* variants on susceptibility to opportunistic infections in African Americans from four HIV/AIDS cohorts. Our population genetic epidemiological data revealed a potential role of *APOL1* variant alleles in protection against AIDS-related opportunistic infections.

A subgroup analysis of the LSOCA cohort revealed that *APOL1* variant alleles are specifically associated with protection against fungal OIs, but not with viral, parasitic, or bacterial OIs. From studying the longitudinal seroconverter ALIVE cohort, we recently reported that *APOL1* variants confer no obvious effect on HIV viral load^11^. This is further confirmed by the results from the seroprevalent patients in the four cohorts included in this study. This suggests that the APOL1 association with OI is unlikely to be mediated by affecting HIV replication but rather, more likely, by inhibiting OI-inducing pathogens directly or via the immune response. The direct inhibition of fungi by overexpression of APOL1 and its variants has recently been demonstrated in vitro^12^. Expression of human APOL1 reduces yeast *S. cerevisiae* growth, through impairment of endosomal trafficking and acidification processes^12^. APOL1 G1 and G2 variant proteins conferred significantly greater toxicity to yeast compared with the wild-type APOL1 G0, likely due to differential impairment of vacuole acidification^12^. The APOL1 G1 and G2 variant proteins kill *T.b. rhodesiense* by evading virulence factor serum resistance-associated protein (SRA) encoded by the *T.b. rhodensiense*^1,10,13,14^. Perhaps, fungi contain a similar counteractive mechanism that differentially interacts with variant APOL1 and APOL1 proteins.

Variant APOL1 may also affect susceptibility to OI through immune activation and enhancement. Host susceptibility to pathogen invasion is strongly determined by the robustness of the innate immune response, as adaptive immune response takes days to develop. APOL1 is upregulated by pro-inflammatory cytokines such as IFN-γ, IFN-β, IFN-α, and TNF, which are induced by invading viruses and other pathogens, including HIV and fungi^15–17^. APOL1 and APOL1 variant protein may differentially induce macrophage polarization and modify immune responses^9^. APOL1, under stimulation of pro-inflammatory cytokines IFNγ and lipopolysaccharide, a major component of the outer membrane of Gram-negative bacteria, induced differentiation of THP-1 monocyte cells into the polarization of atypical M1 macrophage state. APOL1 G1 and G2 variants induced more *IL6* and *TNF* mRNA (M1 marker), a presumably stronger M1 state, compared with APOL1-G0 protein that carried no variant alleles^9^. Macrophages are among the first-line effectors of the innate immune defense against invading pathogens^15,18,19^. Activated M1 macrophages perform microbicidal function through phagocytosis of internalized pathogens and producing cytokines and chemokines to recruit other immune cells to control the infection^18–20^. Together, these data support differential roles of APOL1 protein isoforms in the immune defense of OI, although the mechanisms remain unresolved.

APOL1 expression is increased by elevated circulating levels of interferon in several clinical settings, leading to glomerular injury. These settings include (1) chronic viral infection, e.g., with HIV infection (as discussed above) and with parvovirus B19 infection^21^, (2) administration of therapeutic interferon, given for other indications^16^, and (3) a genetic disorder, e.g., the stimulator of interferon-gene (STING)-associated vasculopathy with onset in infancy (SAVI). It is likely that this list will grow longer.

The study adds to evidence that APOL1 or its variant isoforms may confer protection to a broader range of pathogens than only African trypanosomes. APOL1 has been shown to confer resistance against *Trypanosoma brucei*^1^, amelioration of Leishmania infection, and inhibition of HIV-1 replication in certain cell types^8^. *APOL1* G1 and G2 variants exhibit recent positive selection signals in the form of extended haplotypes in some West Africa populations^2,4^, possibly due to selective pressure from pathogens during recent evolution^14^. The broader role of APOL1 as an innate immune factor against fungal pathogens, if validated, may explain in part why *APOL1* G1 and G2 variants have been selected in African-ancestry populations, despite the increased risk for kidney disease and preeclampsia^22^. A protective impact of *APOL1* variant alleles against OIs could therefore influence HIV disease outcomes among African-ancestry populations and have implications for targeted management. It is possible that *APOL1* G1 and G2 variants could be under positive selective pressure from OIs, in addition to *T.b. rhodesiense* and *T.b. gambiense*. In addition to pleiotropic associations of *APOL1* risk alleles with human African trypanosomiasis, kidney disease, cardiovascular disease, and preeclampsia, carriage of *APOL1* risk alleles was unexpectedly found to be associated with an elevated risk of sepsis in a study of older, community-dwelling black participants enrolled in the REGARDS (reasons for geographic and racial differences in stoke)^23^. This is consistent with our observation of *APOL1* risk alleles increasing the risk of bacterial pneumonia, as most sepsis is caused by bacterial infections. *APOL1* variants may have pleiotropic-modifying effects on innate immune response or inflammatory responses to different classes of human pathogens.

There are limitations to our study. The strength of statistical association for OI is modest or nonsignificant in individual cohorts, likely due to the relatively low frequency of *APOL1* variant alleles and the modest sample size. With the sample size in the combined cohorts, assuming a 30% OI prevalence rate, we had 80% power to detect a protective effect of OR of ≥0.43, but only 30% power to detect the observed effect size on OI (OR of 0.60). Nevertheless, *APOL1* variant- protective effect on OI was largely consistent in the direction across the cohorts with diverse demographical features and at various disease stages, supporting the observed association. Although we only observed a protective effect with two copies of *APOL1* variant alleles, we cannot exclude the possibility that an additive effect would be revealed with larger sample size. Similarly, *APOL1* variants increase renal disease risk in the recessive model, possibly because a critical threshold of APOL1 protein level is required for cell toxicity^24,25^. Another concern is that *APOL1* carriers of two risk alleles who developed HIV-associated nephropathy (HIVAN) may not have been enrolled in the seroprevalent cohorts. We also had the potential for incomplete data for prevalent OI diagnoses. A limitation of our study is that we only have data on the presence/absence of OI, but not OI disease severity. The association of the G1 and G2 alleles with trypanosome susceptibility and trypanosomiasis disease severity differs by trypanosome strain and by APOL1 G1 or G2^6^; therefore, it is possible that the effect of APOL1 variants on infection susceptibility and disease severity may differ by OI and *APOL1* genotype.

This study should be considered as exploratory and hypothesis-generating. More studies with a larger sample size are required to reach a definitive conclusion and to elucidate specific mechanisms leading to protection against OI. It also remains to be determined if the APOL1 association with OI in the settings of HIV infection extends to other settings of immune suppression (e.g., individuals with nephrotic syndrome or chronic and end-stage kidney disease experiencing uremia, or transplant recipients and cancer patients taking immunosuppressive drugs).

In summary, this population genetic study suggested that *APOL1* might confer carriers of two variant alleles’ protection from HIV-related opportunistic infections, especially fungal infections. These findings warrant further replication and experimental validation and extension to infectious disease incidence and prevalence in populations of recent African ancestry, particularly those with chronic kidney disease and end-stage kidney disease and those immunocompromised due to many other diseases.

## Methods

### Ethics statement

Ethical approval for the study was obtained from the National Institute of Health Office of Human Subjects Research Protections (OHSRP #3314). Institutional Review Boards of all participating institutions approved the study protocols and written informed consent was obtained from all study participants.

### Study participants

We studied African American subjects enrolled in four US-based HIV cohorts since *APOL1* G1–G2 alleles are only present in individuals with recent African ancestry. The four HIV cohorts include the ALIVE, consisting of half seroconverters and half of the seroprevalence, and the seroprevalent cohorts LSOCA, MACS, and WIHS.

#### The ALIVE cohort

The epidemiological and clinical characteristics of the ALIVE (AIDS link to the intravenous experience) cohort have been previously described^26^. ALIVE is a prospective longitudinal cohort originally designed to characterize the incidence and natural history of HIV infection among injection drug users (IDU) in Baltimore, MD, initiated in 1988^26^. The participants were followed at six-month intervals with blood draws for viral load and CD4+ T-cell measurements and physical exam at each visit. The censoring date used was the date of the last recorded visit, if prior to July 21, 1997, otherwise a date of July 31, 1997, was used, in order to minimize the confounding effect of antiretroviral therapy (ART)^27^. The study group includes 227 African American incident HIV seroconverters and 213 HIV-seroprevalent individuals (acquired HIV prior to study entry).

#### The LSOCA cohort

The LSOCA (longitudinal study or the ocular complications of AIDS) was a multicenter prospective observational study of patients diagnosed with AIDS^28^. The study was originally designed for the the occurrence and consequences of ocular opportunistic infections, particularly cytomegalovirus (CMV) retinitis, among patients with AIDS. Participants were enrolled at 19 clinical centers throughout the United States in 1998–2011. Each patient gave a detailed medical and HIV-related disease history and relevant findings were confirmed from the medical records. At least every 6 months, patients were examined and the standardized data were collected on AIDS history and treatment, eye examinations, and hematologic, virologic, and immunologic laboratory data^29–31^. Only baseline data at study entry were used in this study, as all these seroprevalent participants (*n* = 784) already had AIDS at the study entry.

#### The MACS cohort

Multicenter AIDS Cohort Study (MACS) is a longitudinal prospective cohort of men who have sex with men from four US cities: Chicago, Baltimore, Pittsburgh, and Los Angeles, with enrollment starting in 1984^32^. Participants were followed at 6-month intervals. The data-censoring date was the earliest of the date of the last recorded visit, or December 31, 1995. In this study, 651 HIV-positive African Americans (90% seroprevalents) with complete *APOL1* genotype, phenotype, and covariate information were included; their enrollment date ranged from 1984 to 2003 with an average follow-up of 10 years.

#### The WIHS cohort

The Women’s Interagency HIV Study (WIHS) is the largest multicenter longitudinal cohort of HIV-positive women in the United States, starting in 1994–1995^33,34^. Participants were seen at 6-month intervals for laboratory and physical examinations. The current analysis included 91 seroprevalent HIV-positive non-Hispanic black women with *APOL1* genotype and OI diagnostic information at study entry.

### Diagnosis of opportunistic infections

AIDS-defining OI was classified based on CDC-revised 1993 AIDS case definition and the MMWR Recommendations^28,35^. The classification of opportunistic infections was made according to the AIDS Clinical Trials Group guidelines. Different etiologies for OIs were available for the LSOCA cohort, allowing us to test for *APOL1* associations for the following subcategories: viral OI (including CMV, Kaposi sarcoma-related herpes virus, and herpes simplex virus), parasitic OIs (including extrapulmonary pneumocystosis, toxoplasma infections, cryptosporidiosis, and isosporiasis), fungal OI (including *Pneumocystis carinii* pneumonia (PCP), candidiasis, cryptococcal, histoplasmosis, and coccidioidomycal infections), and bacterial OI (including *Mycobacterium tuberculosis*, *Mycobacterium avium* complex (MAC), *Mycobacterium kansasii*, *Mycobacterium genovensii*, and other mycobacterial infections).

### Genotyping of APOL1 G1–G2 variant alleles

*APOL1* coding variants G1 (rs73885319, p.S342G) and G2 (rs71785313, p.N388_Y389del) were genotyped using ABI TaqMan genotyping assays on an ABI 7900HT sequencer detector system (Applied Biosystems, Foster City, CA), as previously described^3^. For quality control, water controls were included on each plate and 10% of samples were duplicated. No water contamination or genotype mismatches between duplicates were observed. G1 and G2 allele calls were also validated in the ALIVE cohort by the Sanger sequencing, following a previously described protocol^36^.

The *APOL1* variants were genotyped in the Winkler lab for the ALIVE^11^ and LSOCA cohorts. The MACS and WIHS participants were genotyped by the cohort studies using Taqman protocols, as previously published^37,38^.

### Defining APOL1 variant alleles

The *APOL1* G1 variant allele is defined by the presence of rs73885319 G1 (342G), which is in almost complete positive linkage disequilibrium with rs60910145 (384M), and the G2 variant allele by rs71785313, an in-frame 6-base deletion (TTATAA), leading to the loss of two amino acids (p.N388_Y389del); the G0 haplotype contains neither the G1 nor the G2 variant allele^2,3^. The G1 and G2 variants are in absolute negative disequilibrium and always occur on different chromosomes^2,3^. Individuals carrying any two variant alleles in the homozygous or compound heterozygous state (G1/G1, G2/G2, or G1/G2) are considered *APOL1* high-risk (HR) carriers and are at increased risk for kidney disease; individuals carrying 0 or 1 risk allele are defined as *APOL1* low-risk carriers (LR) for kidney disease^37^.

### Statistical analysis

We evaluated the effects of *APOL1* variant alleles using an additive model (2 vs. 1 vs. 0 copies), a dominant model (2 or 1 copies vs. 0 copies), and a recessive model (2 vs. 1 or 0 copies). Analyses were performed using SAS version 9.12 (SAS Institute, Cary, NC). We tested Hardy–Weinberg equilibrium (HWE) of *APOL1* variant genotypes by using a goodness-of-fit *χ*^2^ test^39^ and an exact test^40^.

We compared the mean baseline CD4 T-cell counts between the group carrying two *APOL1* variant alleles and the group carrying 1 or 0 variant alleles using ANOVA. We adjusted the regression model analyses by sex and by age at seroconversion, or age at study entry for those who were seroprevalent, using the following age categories: 0–19, 20–40, and >40 years.

To account for potential population stratification among participants, we adjusted the regression model association tests in the ALIVE and WIHS cohort using the first five eigenvalues generated with principal component analysis (PCA) implemented in Eigenstrat^41^, using African-ancestry informative markers^42^ or GWAS data^43^; the PCA data were not available for the particular LSOCA and MACS datasets used in this study.

#### Analysis of opportunistic infections (OIs)

We evaluated the impact of *APOL1* variant alleles on OI acquisition by comparing the frequencies of *APOL1* genotypes between those with OI and those without OI among all HIV-positive subjects in the ALIVE (including seroconverters and seroprevalent subjects) using OI outcomes at the cohort- censoring date. For the WIHS and LSOCA cohorts, OI status at study enrollment was used to minimize the influence of ART and prophylaxis on OI outcomes. Odds ratios (OR) and two-tailed *P* values were obtained by chi-square tests or using a conditional logistic regression model. The regression model was adjusted for age, sex, HIV-1 viral load, ART use, and OI prevention medications, and proportions of African ancestry using the first five principal components (PC), based on the data available from each cohort. We obtained the Bonferroni multiple testing-corrected *P* value with p.adjust function from the stats package in R.

#### Meta-analysis

Meta-analysis was performed by calculating the inverse variance of OR and 95% CI in a random-effects model as implemented in the RevMan V.5.3 software (Cochrane Community, Copenhagen)^44–47^. Statistical heterogeneity between studies was assessed by calculating *tau*-squared (τ^2^), chi-squared (*χ*^2^) test, *P* values, and *I*^2^. Under a random-effects model in the meta-analysis, the variance of the distribution of true effect sizes was estimated by τ^2^. A low *P* value provides evidence of variation in effect estimates beyond chance. The *I*² statistic describes the fraction of variance across studies that is due to heterogeneity^44,45^.

### Reporting summary

Further information on research design is available in the Nature Research Reporting Summary linked to this article.

## Supplementary information

Supplementary Information

Reporting Summary

## Data Availability

The genotype data of HIV seroconverters in the ALIVE cohort were previously reported^11^ and can be accessed at doi: 10.3389/fimmu.2019.00053. All other data that support the findings of this study are included in this published article and its Supplementary Information files or are available from the corresponding authors on reasonable request.
