# Supplementary material for: APOL1 variant alleles associate with reduced risk for opportunistic infections in HIV infection
Source: Commun Biol. 2021 Mar 5;4:284. doi: 10.1038/s42003-021-01812-z (PMC7977062; doi:10.1038/s42003-021-01812-z)
Supplement: Supplementary file 1 — Supplementary Information [file 42003_2021_1812_MOESM1_ESM.pdf]

**Supplementary Table 1. Association of carriage of 2 versus 1 or 0 *APOL1* variant alleles with opportunistic infection in HIV cohorts, leave-one-out metaanalysis.**

| Cohort combination | *APOL1* 2 variant alleles carriers/OI+. N (%) | *APOL1* 2 variant alleles carriers/OI-. N (%) | *APOL1* 2 variant alleles carriers OR (95% CI) | *P* |
|---|---|---|---|---|
| ALIVE+LSOCA+MACS | 52/697(7.5) | 154/1178 (13.07) | 0.62 (0.43-0.89) | 0.01 |
| ALIVE+MACS+WIHS | 37/372 (9.9) | 130/911 (14.3) | 0.55 (0.35-0.88) | 0.01 |

Note: Results were from a metaanalysis using RevMan 5. For more details and the LSOCA+MACS+WIHS cohort combination, see Table 3.

**Supplementary Table 2. Association of two *APOL1* G1-G2 alleles (recessive genetic model) with most common baseline AIDS-defining opportunistic infections among LSOCA participants**

| Infection | N | *APOL1* 2 variant alleles carriers /case | *APOL1* 2 variant alleles carriers /control | OR | *P* |
|---|---|---|---|---|---|
| Fungal Pneumocystis carinii pneumonia | 274 | 16/248 | 49/536 | 0.67 | 0.18 |
| Fungal Esophageal candidiasis | 149 | 7/133 | 58/651 | 0.54 | 0.13 |
| Mucocutaneous herpes simplex (HSV) | 78 | 6/70 | 59/714 | 1.12 | 0.93 |
| Mycobacterium avium complex | 66 | 1/53 | 64/731 | 0.19 | 0.10 |
| Bacterial pneumonia | 60 | 9/56 | 56/728 | 2.54 | 0.03 |
| Fungal Cryptococcal meningitis | 51 | 0/47 | 65/737 | 0[a] | 0.03 [a] |
| Localized Herpes Zoster | 47 | 4/43 | 61/741 | 1.25 | 0.68 |

Note: OR and *P* values are for multivariate models adjusted for HAART, age, sex, HIV transmission routes, and HIV load except for Cryptococcal meningitis.

[a]None of patients with two *APOL1* G1-G2 alleles had Cryptococcal meningitis infections, therefore multivariate regression models could not be estimated and OR and *P* values were from Fisher's exact test.

**Supplementary Table 3. Number of multiple opportunistic infections stratified by *APOL1* G1-G2 alleles**

| **Number of *APOL1* G1-G2 variant alleles** | **Median (25%,75%) number of OIs** | **Mean number of OIs** |
|---|---|---|
| 0 | 2 (0,3) | 2.23 |
| 1 | 2 (0,3) | 2.11 |
| 2 | 2 (0,2) | 1.59 |
| | | |
| All patients | 2 (0,4) | 2.16 |
| Multivariate Regression model *(*APOL1* recessive) | | beta=-0.58±0.27, *P*=0.03 |

***** Model adjusted for HAART, age, sex, HIV transmission route, and HIV load.
