# Supplementary material for: APOL1 variant alleles associate with reduced risk for opportunistic infections in HIV infection
Source: Commun Biol. 2021 Mar 5;4:284. doi: 10.1038/s42003-021-01812-z (PMC7977062; doi:10.1038/s42003-021-01812-z)
Supplement: Supplementary file 2 — Reporting Summary [file 42003_2021_1812_MOESM2_ESM.pdf]

nature research

Corresponding author(s): Ping An

Last updated by author(s): Jan 19, 2021

Reporting Summary

Nature Research wishes to improve the reproducibility of the work that we publish. This form provides structure for consistency and transparency in reporting. For further information on Nature Research policies, see our Editorial Policies and the Editorial Policy Checklist.

Statistics

For all statistical analyses, confirm that the following items are present in the figure legend, table legend, main text, or Methods section.

n/a	Confirmed	
☐	☒	The exact sample size (n) for each experimental group/condition, given as a discrete number and unit of measurement
☐	☒	A statement on whether measurements were taken from distinct samples or whether the same sample was measured repeatedly
☐	☒	The statistical test(s) used AND whether they are one- or two-sided<br>*Only common tests should be described solely by name; describe more complex techniques in the Methods section.*
☐	☒	A description of all covariates tested
☐	☒	A description of any assumptions or corrections, such as tests of normality and adjustment for multiple comparisons
☐	☒	A full description of the statistical parameters including central tendency (e.g. means) or other basic estimates (e.g. regression coefficient) AND variation (e.g. standard deviation) or associated estimates of uncertainty (e.g. confidence intervals)
☐	☒	For null hypothesis testing, the test statistic (e.g. F, t, r) with confidence intervals, effect sizes, degrees of freedom and P value noted<br>*Give P values as exact values whenever suitable.*
☒	☐	For Bayesian analysis, information on the choice of priors and Markov chain Monte Carlo settings
☐	☒	For hierarchical and complex designs, identification of the appropriate level for tests and full reporting of outcomes
☐	☒	Estimates of effect sizes (e.g. Cohen's d, Pearson's r), indicating how they were calculated

Our web collection on statistics for biologists contains articles on many of the points above.

Software and code

Policy information about availability of computer code

Data collection	Commercial and open source software names and versions were described in the manuscript
Data analysis	Standard analysis methods in Commercial and open source softwares and versions were described in the manuscript

For manuscripts utilizing custom algorithms or software that are central to the research but not yet described in published literature, software must be made available to editors and reviewers. We strongly encourage code deposition in a community repository (e.g. GitHub). See the Nature Research guidelines for submitting code & software for further information.

Data

Policy information about availability of data

All manuscripts must include a data availability statement. This statement should provide the following information, where applicable:

- Accession codes, unique identifiers, or web links for publicly available datasets
- A list of figures that have associated raw data
- A description of any restrictions on data availability

The data generated or analysed during this study are included in this published article and its supplementary information files or are available from the corresponding authors on reasonable request

Field-specific reporting

Please select the one below that is the best fit for your research. If you are not sure, read the appropriate sections before making your selection.

☒ Life sciences ☐ Behavioural & social sciences ☐ Ecological, evolutionary & environmental sciences

For a reference copy of the document with all sections, see nature.com/documents/nr-reporting-summary-flat.pdf

Life sciences study design

All studies must disclose on these points even when the disclosure is negative.

Sample size	We maximized the sample size by combining 4 cohorts of Africa Americans, practically possible . By power calculation we had 80% power to detect a protective effect of OR of ≥0.43, though we admitted in the limitation that inadequate power for smaller effect size.
Data exclusions	All available patients with clinical data and genotypes were included in the analysis
Replication	The result from discovery cohort led to replication studies in 3 additional cohorts and meta-analysis was conducted.
Randomization	Randomization is not applicable to this study. Relevant covariates were used in the analyses.
Blinding	Clinical data were collected by national-wide cohort clinicians and investigators, genotyping was done by Technicians at Frederick National Lab, and analysts were independent from data collection and data generation.

Reporting for specific materials, systems and methods

We require information from authors about some types of materials, experimental systems and methods used in many studies. Here, indicate whether each material, system or method listed is relevant to your study. If you are not sure if a list item applies to your research, read the appropriate section before selecting a response.

Materials & experimental systems

n/a	Involved in the study	
☒	☐	Antibodies
☒	☐	Eukaryotic cell lines
☒	☐	Palaeontology and archaeology
☒	☐	Animals and other organisms
☐	☒	Human research participants
☒	☐	Clinical data
☒	☐	Dual use research of concern

Methods

n/a	Involved in the study	
☒	☐	ChIP-seq
☒	☐	Flow cytometry
☒	☐	MRI-based neuroimaging

Human research participants

Policy information about studies involving human research participants

Population characteristics	Population characteristics such as age, gender, genotypes, treatment and diagnostic were used as covariates in the analysis.
Recruitment	Participants were recruited for the natural history and treatment cohorts with pre-defined criteria, which have been well-described.
Ethics oversight	NIH and participating Universities approved the study protocol, as described in the manuscript

Note that full information on the approval of the study protocol must also be provided in the manuscript.
